# Supplementary material for: Cerebral microstructural alterations in Post-COVID-condition are related to cognitive impairment, olfactory dysfunction and fatigue
Source: Nat Commun. 2024 May 18;15:4256. doi: 10.1038/s41467-024-48651-0 (PMC11102465; doi:10.1038/s41467-024-48651-0)
Supplement: Supplementary file 1 — Supplementary Information [file 41467_2024_48651_MOESM1_ESM.pdf]

Cerebral microstructural alterations in Post-COVID-condition are related to cognitive impairment, olfactory dysfunction and fatigue

Jonas A Hosp[1*], Marco Reisert[2,3*], Andrea Dressing[1,4], Veronika Götz[5], Elias Kellner[2], Hansjörg Mast[6], Susan Arndt[7], Cornelius F. Waller[8], Dirk Wagner[5], Siegbert Rieg[5], Horst Urbach[6], Cornelius Weiller[1], Nils Schröter[1*], Alexander Rau[6*]

1: Department of Neurology and Clinical Neuroscience, Medical Center – University of Freiburg, Faculty of Medicine, University of Freiburg, Freiburg, Germany

2: Department of Diagnostic and Interventional Radiology, Medical Center – University of Freiburg, Faculty of Medicine, University of Freiburg, Freiburg, Germany

3: Department of Stereotactic and Functional Neurosurgery, Medical Center – University of Freiburg, Faculty of Medicine, University of Freiburg, Freiburg, Germany

4: Freiburg Brain Imaging Center, Medical Center – University of Freiburg, Faculty of Medicine, University of Freiburg, Freiburg, Germany

5: Department of Internal Medicine II, Medical Center – University of Freiburg, Faculty of Medicine, University of Freiburg, Freiburg, Germany

6: Department of Neuroradiology, Medical Center – University of Freiburg, Faculty of Medicine, University of Freiburg, Freiburg, Germany

7: Department of Otorhinolaryngology ‑ Head and Neck Surgery, Medical Center ‑ University of Freiburg, Faculty of Medicine, University of Freiburg

8: Department of Internal Medicine I, Medical Center ‑ University of Freiburg, Faculty of Medicine, University of Freiburg

* These authors contributed equally to this work.

Corresponding author:
Jonas A. Hosp MD
E-mail: jonas.hosp@uniklinik-freiburg.de
University Medical Center Freiburg
Department of Neurology and Neuroscience
Breisacher Str. 64, 79106 Freiburg, Germany
phone:+49 761 270 50010
fax:+49 761 270 53100
ORCID: 0000-0001-7696-9334

Running title: Microstructural alterations in Post-COVID-condition

Key Words: Diffusion Microstructure Imaging, COVID-19, Post-COVID-condition, cognition, fatigue, olfaction

Supplementary Figures

Gray-matter volume shifts in PCC vs. HNC are similar across periods

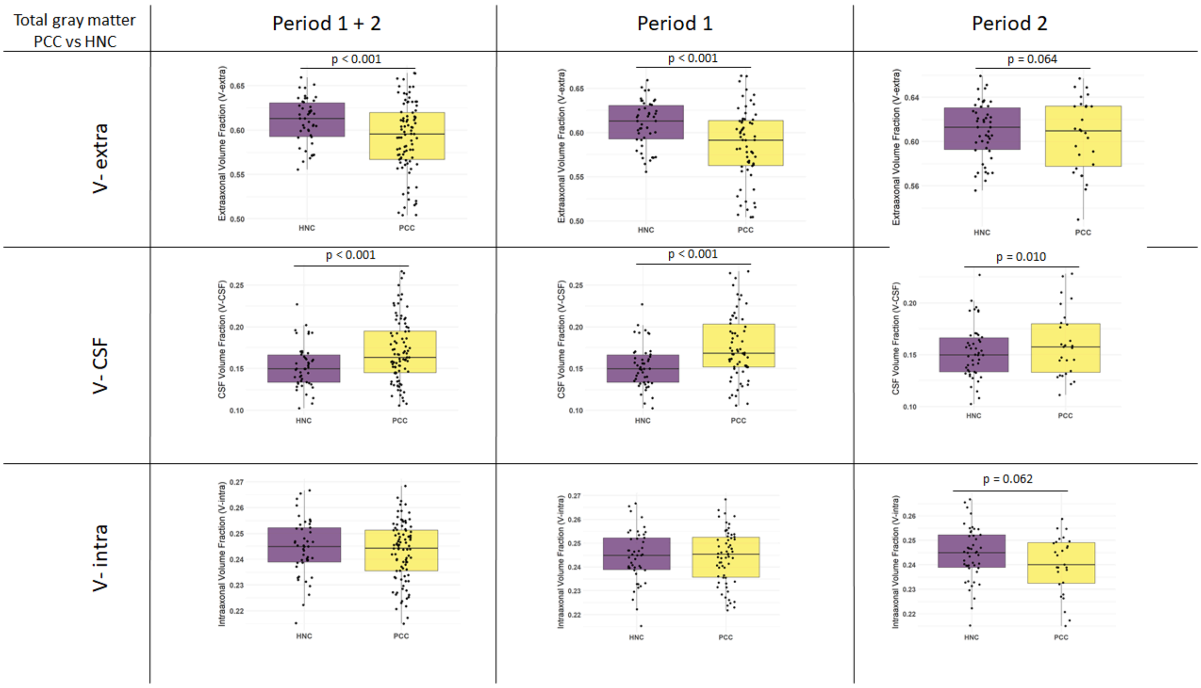

Supplementary Figure 1. Patients with Post-COVID-Condition (PCC) were recruited in two distinct periods. To evaluate a potential influence of this issue, we conducted the comparison of whole-brain gray-matter diffusion MRI parameters with Healthy Non-COVID (HNC) participants separately for both periods. For this purpose, we employed two-tailed ANCOVAs with age and sex as nuisance covariates. Box plots show the distribution of microstructural compartments within the entire gray matter. The center lines represent the median, box bounds show interquartile range (IQR), whiskers cover the data range from minimum to maximum, and dots represent individual subject values. In patients with PCC, a decrease in the extraneurite volume fraction (V-extra) was accompanied by a significant increase in the free-fluid fraction (V-CSF) when compared to the HNC group. This effect persists when analyses were computed separately for period 1 and 2 PCC patients. The statistic was based on 46 HNC and 89 PCC cases (n = 62 in period 1; n = 27 in period 2). Although the significance level in period 2 is lowered due to the lower number of patients (n=27), effect directions were similar with a reduction of V-extra and an increase in V-CSF when compared to HNC-controls.

Gray-matter volume shifts in PCC vs. UPC are similar across periods

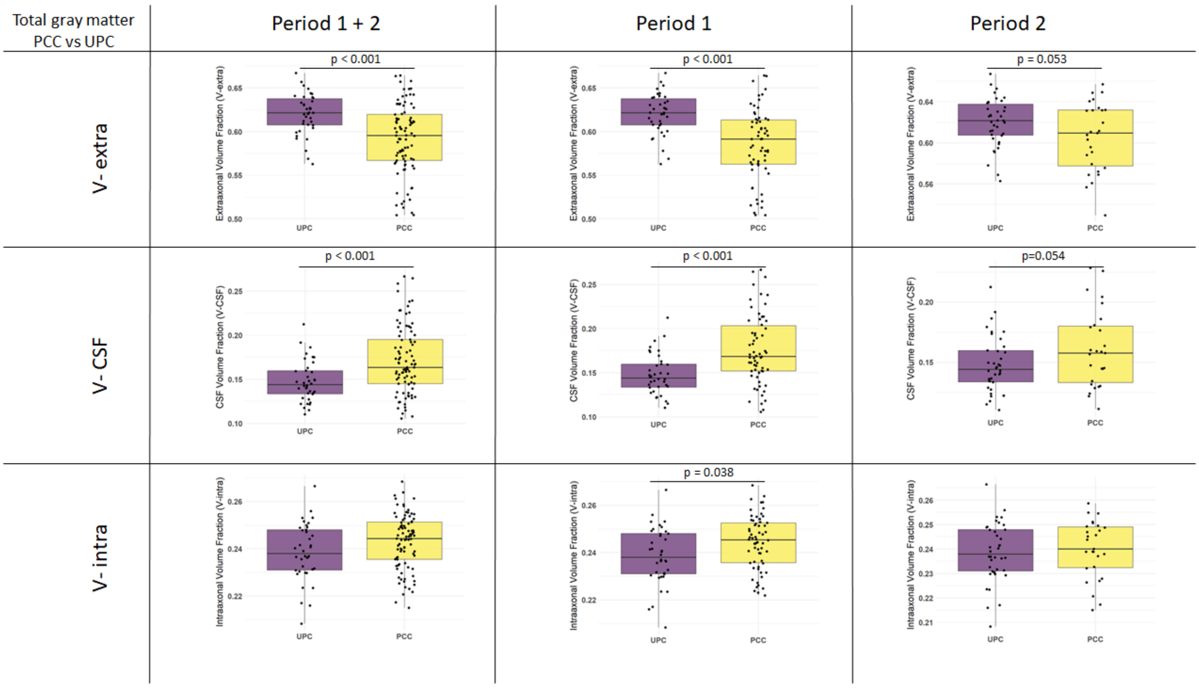

Supplementary Figure 2. Patients with Post-COVID-Condition (PCC) were recruited in two distinct periods. To evaluate a potential influence of this issue, we conducted the comparison of whole-brain gray-matter diffusion MRI parameters with Unimpaired Post-COVID (UPC) participants separately for both periods. For this purpose, we employed two-tailed ANCOVAs with age and sex as nuisance covariates. Box plots show the distribution of microstructural compartments within the entire gray matter. The center lines represent the median, box bounds show interquartile range (IQR), whiskers cover the data range from minimum to maximum, and dots represent individual subject values. In patients with PCC, a decrease in the extraneurite volume fraction (V-extra) was accompanied by a significant increase in the free-fluid fraction (V-CSF) when compared to the UPC-group. This effect persists when analyses were computed separately for period 1 and 2 PCC patients. The statistic was based on 38 UPC and 89 PCC cases (n = 62 in period 1; n = 27 in period 2). Although the significance level in period 2 is lowered due to the lower number of patients with PCC (n=27), effect directions were similar with a reduction of V-extra and an increase in V-CSF when compared to UPC participants.

Disease severity does not affect the spatial distribution of V-extra changes

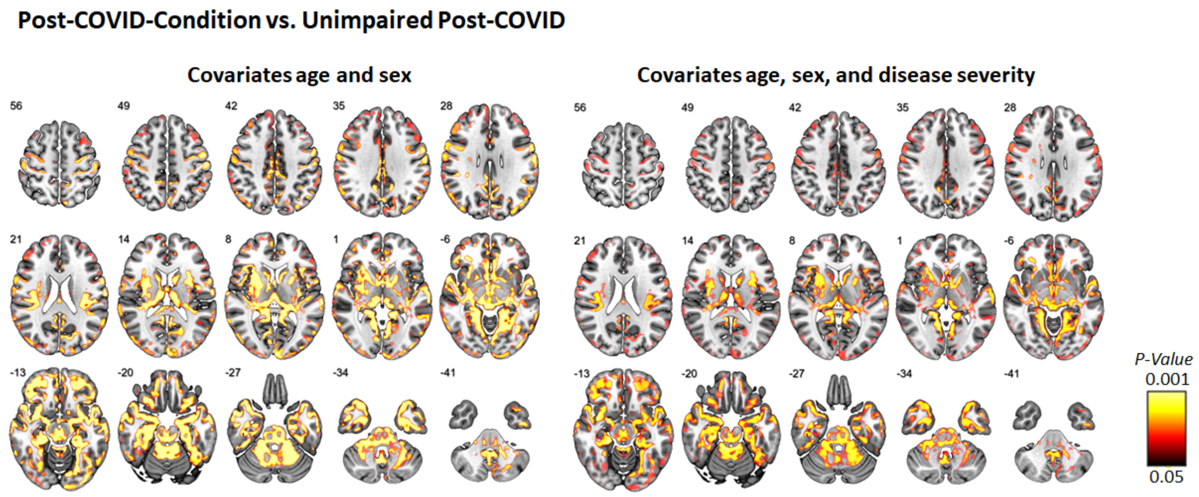

Supplementary Figure 3. To relate clinical outcomes to V-extra in patients with a previous COVID-19 infection (i.e. combined PCC- and UPC-group), voxel-based two-tailed linear regression models were employed with V-extra as a dependent variable, covariates age and sex, and threshold-free cluster enhancement. P-values were corrected for multiple comparisons across voxels using the family-wise error rate (FWE). Voxels with significantly different V-extra are indicated by hot shading and superimposed onto a T1w MRI template. Radiological orientation, i.e. left side of the image corresponds to the right side of the patient's body; numbers denote the axial (*z*) position in millimeters. The statistic was based on 38 UPC and 89 PCC cases. Although disease severity ("grading") significantly affected whole gray matter V-extra, its implementation into the model as covariate did not change the spatial distribution of significant V-extra changes between the PCC- and the UPC-group.

Delay does not affect spatial distribution of significant V-extra changes

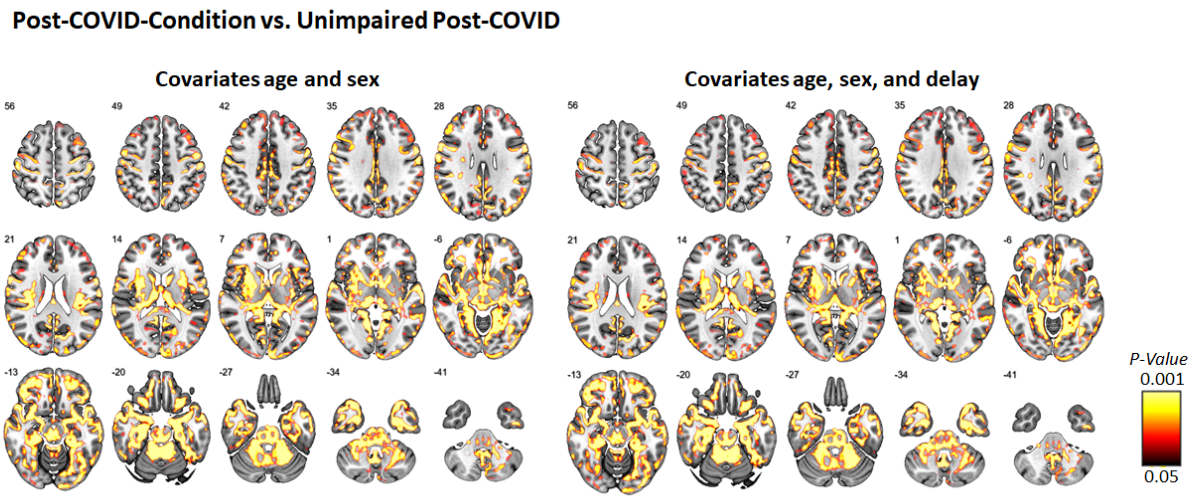

Supplementary Figure 4. To relate clinical outcomes to V-extra in patients with a previous COVID-19 infection (i.e. combined PCC- and UPC-group), voxel-based two-tailed linear regression models were employed with V-extra as a dependent variable, covariates age and sex, and threshold-free cluster enhancement. P-values were corrected for multiple comparisons across voxels using the family-wise error rate (FWE). Voxels with significantly different V-extra are indicated by hot shading and superimposed onto a T1w MRI template. Radiological orientation, i.e. left side of the image corresponds to the right side of the patient's body; numbers denote the axial (*z*) position in millimeters. The statistic was based on 38 UPC and 89 PCC cases. Implementation of the "delay" in days between positive SARS-CoV-2 and MRI-scan into the model as a nuisance covariate did not change the spatial distribution of significant V-extra changes between the PCC- and the UPC-group.

Adding patients of period 2 did not influence effect-patterns (PCC vs. HNC)

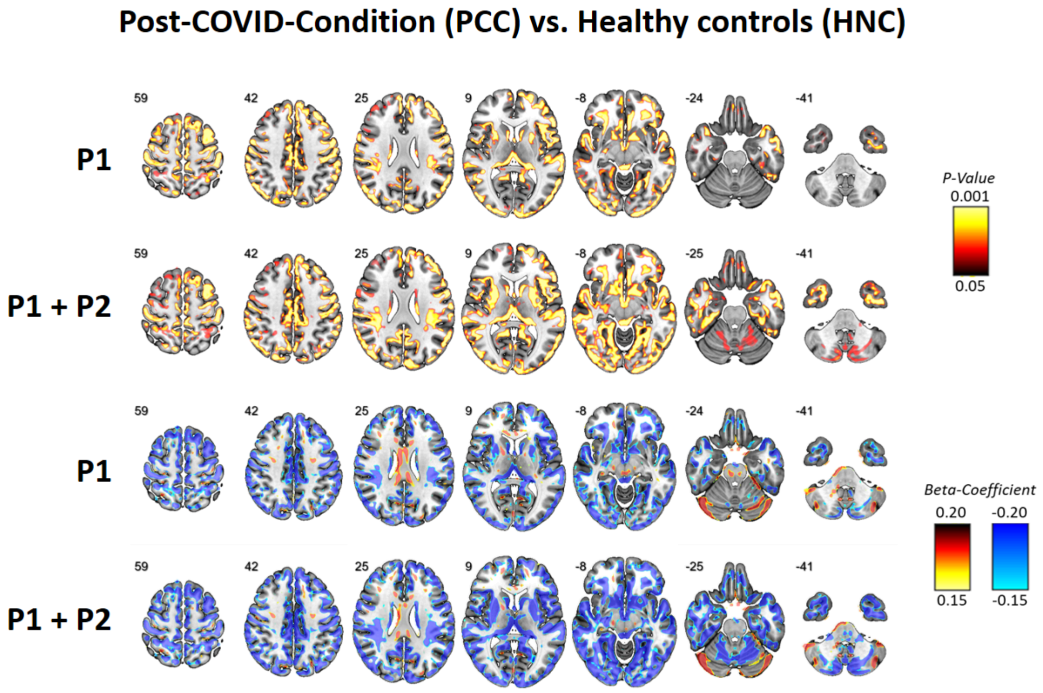

Supplementary Figure 5. Patients with Post-COVID-Condition (PCC) were recruited in two distinct periods. Due to the small number of PCC patients in period 2, we decided against carrying out voxel-based analysis with this particular group alone. Instead, we compared PCC-patients enrolled in period 1 with the combined collective (i.e. period 1 + 2) to test if the inclusion of the additional patients led to any changes. **Top:** Results of statistical voxel-wise two-tailed linear regression models of V-extra after threshold-free cluster enhancement and family-wise-error (FWE)-correction between different groups: Post-COVID-Condition (PCC) vs. Healthy Non-COVID controls (HNC). Voxels with significantly different V-extra were indicated by shading and superimposed onto a T1w MRI template (top rows). P-values were corrected for multiple comparisons across voxels using the family-wise error rate (FWE), and age and sex served as nuisance covariates. Radiological orientation: left side of the image corresponds to the patient's right; numbers denote the axial (*z*) position in millimeters. P1 comprises PCC patients enrolled in period 1, P1 + 2 the entire collective of n = 89 patients. **Bottom:** Results of standardized regression coefficients derived from the same model as indicated above between different groups: Post-COVID-Condition (PCC) vs. Healthy Non-COVID controls (HNC). Color-coding indicates the beta coefficient values as a measure of effect size of the factor COVID-19 (hot colors: positive effects vs. cold colors: negative effects; bottom rows). Radiological orientation: left side of the image corresponds to the patient's right; numbers denote the axial (*z*) position in millimeters. The statistic was based on 46 HNC and 89 PCC cases (n = 62 in period 1; n = 27 in period 2). Thus, adding the PCC-patients enrolled in period 2 did not change spatial patterns of significant effects or effect size/directions expressed by beta-coefficients for PCC vs. HNC group.

Adding patients of period 2 did not influence effect-patterns (PCC vs. UPC)

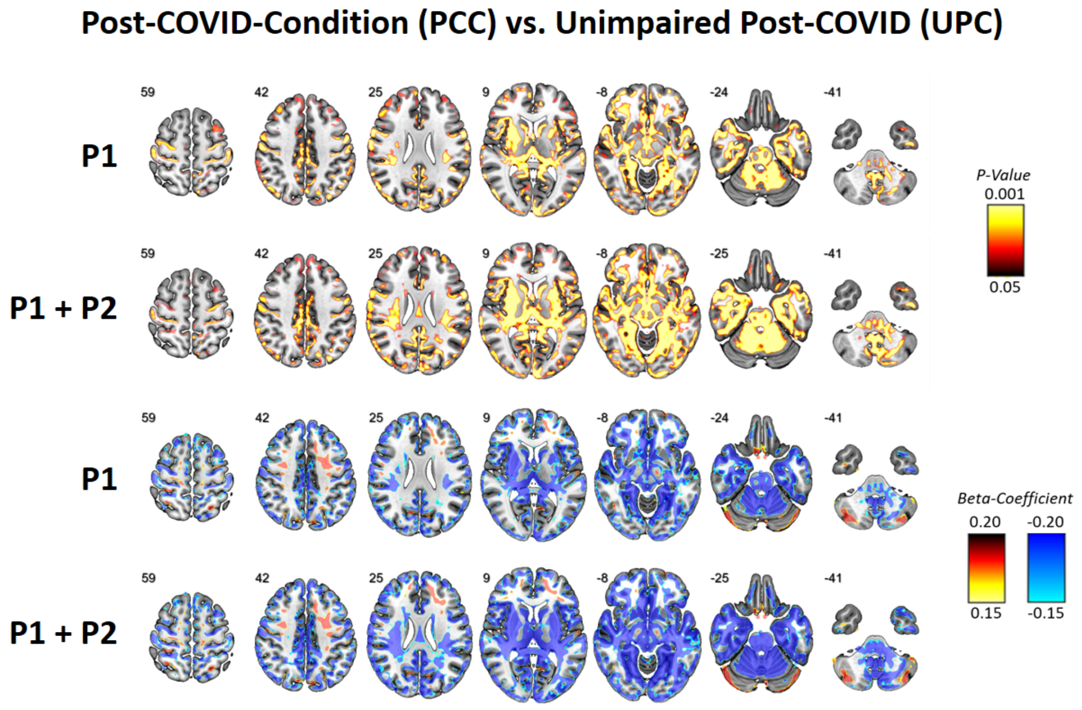

Supplementary Figure 6. Patients with Post-COVID-Condition (PCC) were recruited in two distinct periods. Due to the small number of PCC patients in period 2, we decided against carrying out voxel-based analysis with this particular group alone. Instead, we compared PCC-patients enrolled in period 1 with the combined collective (i.e. period 1 + 2) to test if the inclusion of the additional patients led to any changes. **Top:** Results of statistical voxel-wise two-tailed linear regression models of V-extra after threshold-free cluster enhancement and family-wise-error (FWE)-correction between different groups: Post-COVID-Condition (PCC) vs. Unimpaired Post-COVID group (UPC). Voxels with significantly different V-extra were indicated by shading and superimposed onto a T1w MRI template (top rows). P-values were corrected for multiple comparisons across voxels using the family-wise error rate (FWE), and age and sex served as nuisance covariates. Radiological orientation: left side of the image corresponds to the patient's right; numbers denote the axial (*z*) position in millimeters. P1 comprises PCC patients enrolled in period 1, P1 + 2 the entire collective of n = 89 patients. **Bottom:** Results of standardized regression coefficients derived from the same model as indicated above between different groups: Unimpaired Post-COVID group (UPC). Color-coding indicates the beta coefficient values as a measure of effect size of the factor COVID-19 (hot colors: positive effects vs. cold colors: negative effects; bottom rows). Radiological orientation: left side of the image corresponds to the patient's right; numbers denote the axial (*z*) position in millimeters. The statistic was based on 38 UPC and 89 PCC cases (n = 62 in period 1; n = 27 in period 2). Thus, adding the PCC-patients enrolled in period 2 did not change spatial patterns of significant effects or effect size/directions expressed by beta-coefficients for PCC vs. UPC group.

Similar pattern for mental and physical domains of fatigue

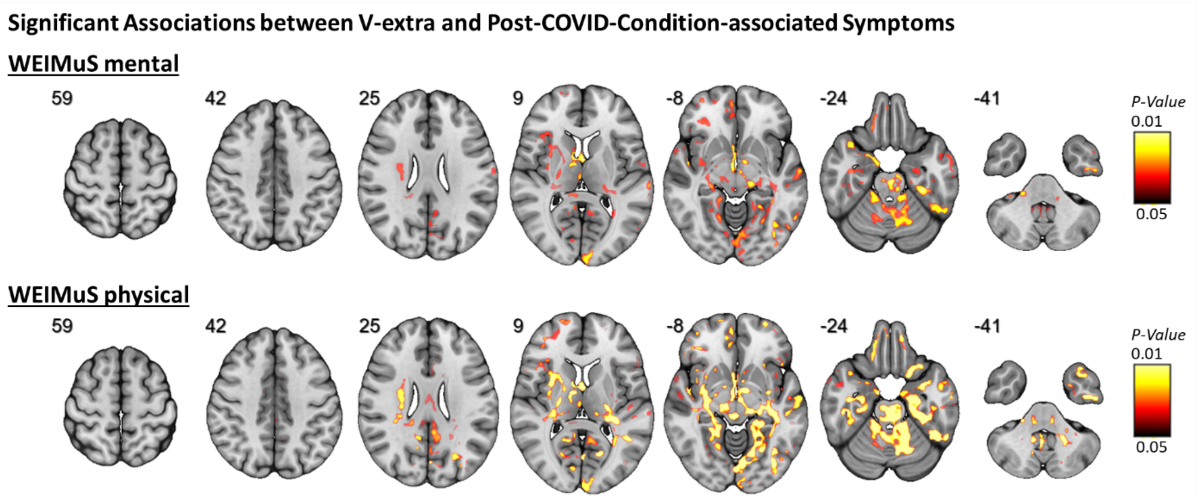

Supplementary Figure 7. To relate clinical outcomes to V-extra in patients with a previous COVID-19 infection (i.e. combined PCC- and UPC-group), voxel-based two-tailed linear regression models were employed, with V-extra as a dependent variable, covariates age and sex, and threshold-free cluster enhancement. P-values were corrected for multiple comparisons across voxels using the family-wise error rate (FWE). Voxels with significantly different V-extra are indicated by hot shading and superimposed onto a T1w MRI template. Radiological orientation, i.e. left side of the image corresponds to the right side of the patient's body; numbers denote the axial (*z*) position in millimeters. This statistic was based on n = 127 patients with previous COVID-19 infection. Detailed investigation of fatigue reveals an almost identical pattern for mental domains of the WEIMuS score as the total score. Also, the physical domains of the WEIMuS revealed a similar emphasis, though the effect was overall stronger and slightly more widespread.

Period does not influence effect-patterns of models for MoCA, Olfaction and WEIMuS

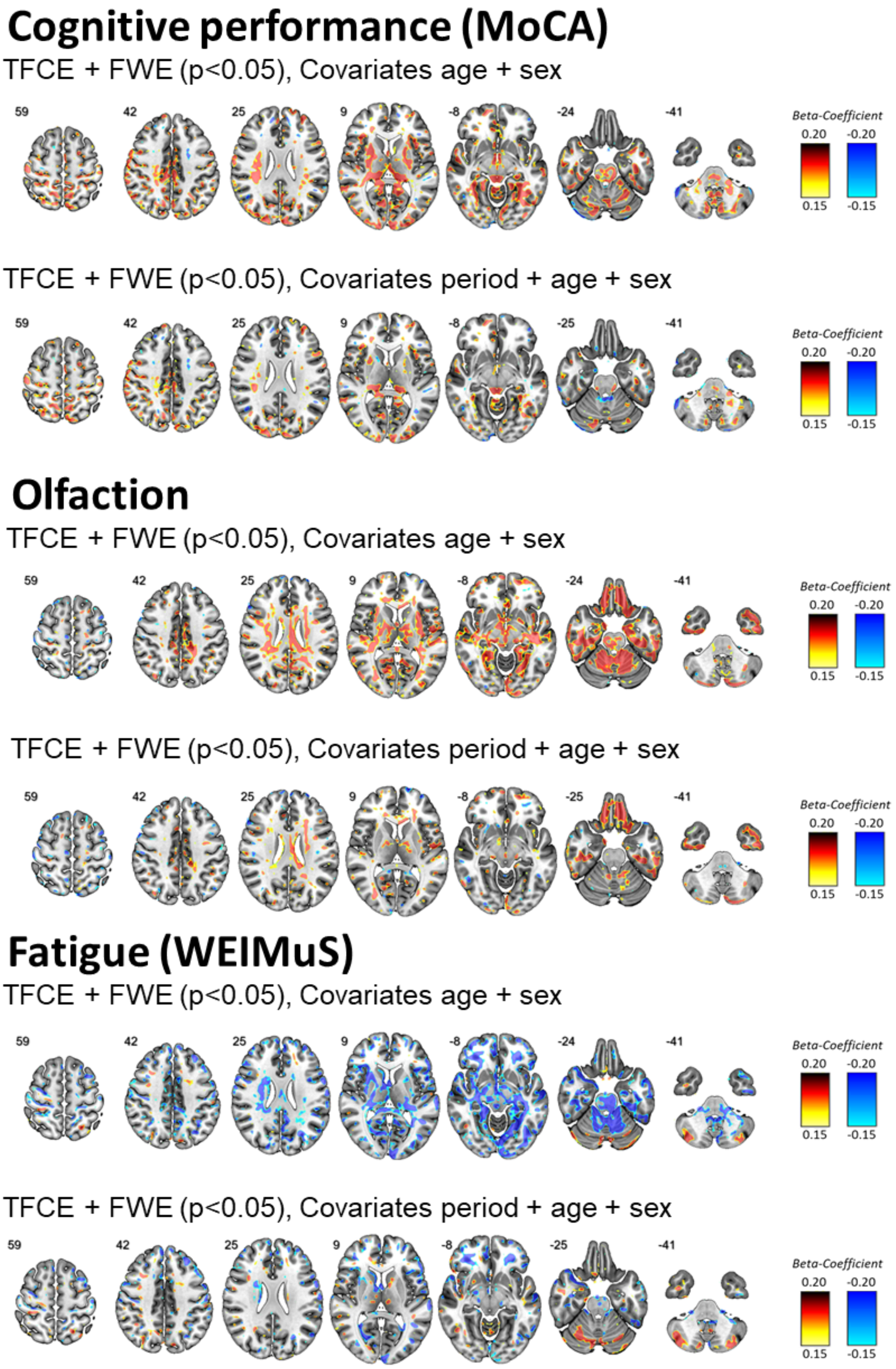

Supplementary Figure 8. Results of standardized regression coefficients derived from voxel-wise two-tailed linear regression voxel-based analyses of associations between clinical scores and V-extra as the dependent variable after TFCE and FWE correction for multiple comparisons. The models were computed with covariates age + sex (respective upper panel) and period + age + sex (respective lower panel). The addition of period did

not influence the characteristic orientations of the symptom-specific patterns. Color-coding indicates the beta coefficient values as a measure of the effect size of the factor COVID-19 (hot colors: positive effects vs. cold colors: negative effects; bottom rows). Radiological orientation: left side of the image corresponds to the patient's right; numbers denote the axial (*z*) position in millimeters. Thus after adding period as a third covariate, effect-patterns were consistent between models for MoCA, Olfaction and WEIMuS, confirming our finding of symptom-specificity of affected networks. This statistic was based on n = 127 patients with previous COVID-19 infection.
