# Supplementary material for: Cerebral microstructural alterations in Post-COVID-condition are related to cognitive impairment, olfactory dysfunction and fatigue
Source: Nat Commun. 2024 May 18;15:4256. doi: 10.1038/s41467-024-48651-0 (PMC11102465; doi:10.1038/s41467-024-48651-0)
Supplement: Supplementary file 3 — Description of Additional Supplementary Files [file 41467_2024_48651_MOESM3_ESM.pdf]

Description of Additional Supplementary Files

File Name: Supplementary Data 1
Description: Detailed version of Table 1: Demographics and comorbidities of study participants

File Name: Supplementary Data 2
Description: Detailed version of Table 2: Clinical characteristics of study participants

File Name: Supplementary Data 3
Description: Results of cortical thickness, surface area, and gray-matter volume analysis (two-sided ANCOVAs controlling for age and sex to assess intergroup differences between PCC, UPC und HNC; FWE-correction for multiple comparisons).

File Name: Supplementary Data 4
Description: Results of total grey-matter volume analysis (two-sided ANCOVAs controlling for age and sex to assess intergroup differences between PCC, UPC und HNC; FWE-correction for multiple comparisons).

File Name: Supplementary Data 5
Description: Results of whole-brain white-matter DMI-parameters (two-sided ANCOVAs controlling for age and sex to assess intergroup differences between PCC, UPC und HNC for V-extra, V-CSF and V-intra; Bonferroni-correction for multiple comparisons).

File Name: Supplementary Data 6
Description: Results of gray-matter DMI parameters with delay as additional nuisance covariate (two-sided ANCOVAs controlling for delay, age and sex to assess intergroup differences between PCC and UPC for V-extra, V-CSF and V-intra; Bonferroni-correction for multiple comparisons).

File Name: Supplementary Data 7
Description: Detailed description of atlas-based anatomic allocations.
