# Supplementary material for: Cerebral microstructural alterations in Post-COVID-condition are related to cognitive impairment, olfactory dysfunction and fatigue
Source: Nat Commun. 2024 May 18;15:4256. doi: 10.1038/s41467-024-48651-0 (PMC11102465; doi:10.1038/s41467-024-48651-0)
Supplement: Supplementary file 5 — Reporting Summary [file 41467_2024_48651_MOESM5_ESM.pdf]

Corresponding author(s): Jonas A. Hosp
Last updated by author(s): 13.03.2024

Reporting Summary

Nature Portfolio wishes to improve the reproducibility of the work that we publish. This form provides structure for consistency and transparency in reporting. For further information on Nature Portfolio policies, see our Editorial Policies and the Editorial Policy Checklist.

Statistics

For all statistical analyses, confirm that the following items are present in the figure legend, table legend, main text, or Methods section.

n/a	Confirmed	
☐	☒	The exact sample size (*n*) for each experimental group/condition, given as a discrete number and unit of measurement
☐	☒	A statement on whether measurements were taken from distinct samples or whether the same sample was measured repeatedly
☐	☒	The statistical test(s) used AND whether they are one- or two-sided<br>*Only common tests should be described solely by name; describe more complex techniques in the Methods section.*
☐	☒	A description of all covariates tested
☐	☒	A description of any assumptions or corrections, such as tests of normality and adjustment for multiple comparisons
☐	☒	A full description of the statistical parameters including central tendency (e.g. means) or other basic estimates (e.g. regression coefficient) AND variation (e.g. standard deviation) or associated estimates of uncertainty (e.g. confidence intervals)
☐	☒	For null hypothesis testing, the test statistic (e.g. *F*, *t*, *r*) with confidence intervals, effect sizes, degrees of freedom and *P* value noted<br>*Give P values as exact values whenever suitable.*
☒	☐	For Bayesian analysis, information on the choice of priors and Markov chain Monte Carlo settings
☒	☐	For hierarchical and complex designs, identification of the appropriate level for tests and full reporting of outcomes
☐	☒	Estimates of effect sizes (e.g. Cohen's *d*, Pearson's *r*), indicating how they were calculated

Our web collection on statistics for biologists contains articles on many of the points above.

Software and code

Policy information about availability of computer code

Data collection	Clinical data was collected in REDCap 14.0.15 (Vanderbilt University) and imaging data was stored in the local PACS (Deep Unity 1.2.0.3. Dedalus, Bonn, Germany).
Data analysis	Data processing was implemented on a local instance of the post-processing platform NORA (www.nora-imaging.org) and CAT12 (http://www.neuro.uni-jena.de/cat/), TFCE (https://github.com/markallenthornton/MatlabTFCE), and FreeSurfer Version 6.0 as well as R (version 4.1.2, https://www.R-project.org/) and SPSS, Version 25 (IBM, Ehningen, Germany).

For manuscripts utilizing custom algorithms or software that are central to the research but not yet described in published literature, software must be made available to editors and reviewers. We strongly encourage code deposition in a community repository (e.g. GitHub). See the Nature Portfolio guidelines for submitting code & software for further information.

Data

Policy information about availability of data

All manuscripts must include a data availability statement. This statement should provide the following information, where applicable:

- Accession codes, unique identifiers, or web links for publicly available datasets
- A description of any restrictions on data availability
- For clinical datasets or third party data, please ensure that the statement adheres to our policy

The anonymized data generated in this study have been deposited in the Dryad database under accession code ZZ [https://doi.org/10.5061/dryad.kkwh70s9g]. As

we did not obtain consent to publish information that identifies individuals, we aggregated age into 5-year categories. The raw MRI data may contain information that could compromise the participants' privacy and can only be made available on request from the corresponding author (JAH, jonas.hosp@uniklinik-freiburg.de; response within four weeks; data might not be used to identify individual participants). The employed cerebral atlases are available via LeadDBS (V2.5; https://www.lead-dbs.org/download/).

Research involving human participants, their data, or biological material

Policy information about studies with human participants or human data. See also policy information about sex, gender (identity/presentation), and sexual orientation and race, ethnicity and racism.

Reporting on sex and gender	In this study, we collected information on participants' sex as self reported and provided within the source data. No information was obtained on gender. To account for potential sex-induced biases, statistical analyses were adjusted for sex as a nuisance covariate.
Reporting on race, ethnicity, or other socially relevant groupings	No socially constructed or socially relevant categorization variable was used in our manuscript.
Population characteristics	89 patients (44 male / 55 female) with a mean age of 49 years were included in the final analyses and compared to 38 subjects after COVID-19 infection without persistent symptoms and 46 healthy controls.
Recruitment	Patients were recruited from the outpatient clinic of the Department of Neurology and Clinical Neuroscience of the University Hospital Freiburg from those who were admitted due to neurocognitive symptoms in the chronic phase of COVID-19 infection. Inclusion criteria were: 1) a SARS-CoV-2 infection confirmed by reverse transcription polymerase chain reaction (rt-PCR); 2) fulfillment of diagnostic criteria for Post-COVID-Condition according to WHO criteria (e.g. >3 months after onset of acute COVID-19 infection; symptoms lasting for at least 2 months; relevant impact on everyday functioning) 3; 3) execution of a cranial MRI. Exclusion criteria were any pre-existing neurodegenerative disorder and an age below 18 years. A potential selection bias is thus conceivable as patients not able to attend the outpatient clinic due to very strong symptoms could not be enrolled.
Ethics oversight	This was approved by the Ethics Committee of the University of Freiburg (EK 211/20).

Note that full information on the approval of the study protocol must also be provided in the manuscript.

Field-specific reporting

Please select the one below that is the best fit for your research. If you are not sure, read the appropriate sections before making your selection.

☒ Life sciences ☐ Behavioural & social sciences ☐ Ecological, evolutionary & environmental sciences

For a reference copy of the document with all sections, see nature.com/documents/nr-reporting-summary-flat.pdf

Life sciences study design

All studies must disclose on these points even when the disclosure is negative.

Sample size	No sample size calculation was carried out in advance. The final sample size was determined by the number of patients referred to our outpatient clinic within the recruitment periods. The final sample size is considered sufficient to statistically support the findings of this study.
Data exclusions	Data from one subject were omitted from further analysis due to MRI-artifacts that would have interfered with further data processing.
Replication	The employed statistical analyses provide identical results upon repetition.
Randomization	As the three groups (i.e. patients with Post-COVID-Condition, patients having contracted COVID-19 without subjective impairment and healthy controls without a history of COVID-19) were defined by the respective diagnose, no randimization was feasible.
Blinding	As the three groups (i.e. patients with Post-COVID-Condition, patients having contracted COVID-19 without subjective impairment and healthy controls without a history of COVID-19) were defined by the respective diagnose, blinding during the analysis was not feasible. Though, as we did not exclude outliers nor investigated individual patients but rather the aforementioned groups, no blinding was necessary for this study design as we did not intestigate therapy options or the diagnostic value of biomarkers.

Reporting for specific materials, systems and methods

We require information from authors about some types of materials, experimental systems and methods used in many studies. Here, indicate whether each material, system or method listed is relevant to your study. If you are not sure if a list item applies to your research, read the appropriate section before selecting a response.

Materials & experimental systems

n/a	Involved in the study
☒	☐ Antibodies
☒	☐ Eukaryotic cell lines
☒	☐ Palaeontology and archaeology
☒	☐ Animals and other organisms
☐	☒ Clinical data
☒	☐ Dual use research of concern
☒	☐ Plants

Methods

n/a	Involved in the study
☒	☐ ChIP-seq
☒	☐ Flow cytometry
☐	☒ MRI-based neuroimaging

Clinical data

Policy information about clinical studies

All manuscripts should comply with the ICMJE guidelines for publication of clinical research and a completed CONSORT checklist must be included with all submissions.

Clinical trial registration: DRKS00021439

Study protocol: https://drks.de/search/de/trial/DRKS00021439

Data collection: Participants with neurocognitive symptoms in the chronic phase after COVID-19 infection were recruited from the outpatient clinic of the Department of Neurology and Clinical Neuroscience of the University Hospital Freiburg between June 16th, 2020, and October 6th, 2022. A potential selection-bias cannot finally be excluded, though we paid special attention on inclusion and exclusion criteria (details given in the Methods).

Outcomes: The reported outcomes were exploratory in nature.

Plants

Seed stocks: N/A

Novel plant genotypes: N/A

Authentication: N/A

Magnetic resonance imaging

Experimental design

Design type: N/A

Design specifications: N/A

Behavioral performance measures: N/A

Acquisition

Imaging type(s): Structual (isotropic 3D T1w, isotropic 3D T2w fluid attenuation inversion recovery) and multishell diffusion-weighted imaging. See Methods for more details.

Field strength: 3

Sequence & imaging parameters: Please see Methods and Rau et al., Brain 2022 for more details.

Area of acquisition: Whole brain

Diffusion MRI: ☒ Used ☐ Not used

Parameters: The diffusion weighted sequence was acquired with the following parameters: axial orientation, 42 slices, voxel size 1.5 x 1.5 x 3

Parameters: mm3, TR 2800 ms, TE 88 ms, bandwidth 1778 Hz, flip angle 90°, simultaneous multi-band acceleration factor 2, GRAPPA factor 2, 58 diffusion-encoding gradient directions with b-factors 1000 and 2000 s/mm2, 15 non-diffusion weighted images (interleaved during diffusion-encoding directions). No cardiac gating was employed.

Preprocessing

Preprocessing software: CAT12 (http://www.neuro.uni-jena.de/cat/); Freesurfer Version 6.0; MatlabTFCE (https://github.com/markallenthornton/MatlabTFCE);

Normalization: Data were spatially normalized by CAT12 using the diffeomorphic anatomical registration using the exponentiated lie algebra (DARTEL) method.

Normalization template: MNI152

Noise and artifact removal: Pre-processing of diffusion-weighted images included a denoising step followed by correction of the Gibbs-ringing artifacts; details are presented in the Methods.

Volume censoring: N/A

Statistical modeling & inference

Model type and settings: Regression/Correlation, further details in Methods.

Effect(s) tested: Effects of SARS-CoV-2 modulated by age and sex (and initial disease severity and delay since infection in auxillary analyses); Correlation of clinical readouts with imaging features modulated by age, sex, time from infection to brainscan and initial disease severity - see Methods for details.

Specify type of analysis: ☐ Whole brain ☐ ROI-based ☒ Both

Anatomical location(s): Anatomical allocation was performed with JHU White Matter Parcellation Map III, FreeSurfer Aparc & Aseg, Human Motor Thalamus, Harvard Ascending Arousal Network. Details are given in the Methods.

Statistic type for inference (See Eklund et al. 2016): N/A

Correction: The family-wise error correction methods was employed to account for multiple testing.

Models & analysis

n/a	Involved in the study
☒	☐ Functional and/or effective connectivity
☒	☐ Graph analysis
☒	☐ Multivariate modeling or predictive analysis
